# Lightweight technology stacks for assistive linked annotations

**DOI:** 10.1186/s44342-024-00021-4

**Published:** 2024-10-10

**Authors:** Nishad Thalhath

**Affiliations:** Laboratory for Large-Scale Biomedical Data Technology, RIKEN Center for Integrative Medical Sciences, Tsurumi, Yokohama, 230-0045 Kanagawa Japan

**Keywords:** Lightweight stacks, JavaScript, Assistive linked annotations, NER, Serverless search, Vector search, Semantic search

## Abstract

This report presents the findings of a project from the 8th Biomedical Linked Annotation Hackathon (BLAH) to explore lightweight technology stacks to enhance assistive linked annotations. Using modern JavaScript frameworks and edge functions, in-browser Named Entity Recognition (NER), serverless embedding and vector search within web interfaces, and efficient serverless full-text search were implemented. Through this experimental approach, a proof of concept to demonstrate the feasibility and performance of these technologies was demonstrated. The results show that lightweight stacks can significantly improve the efficiency and cost-effectiveness of annotation tools and provide a local-first, privacy-oriented, and secure alternative to traditional server-based solutions in various use cases. This work emphasizes the potential of developing annotation interfaces that are more responsive, scalable, and user-friendly, which would benefit bioinformatics researchers, practitioners, and software developers.

## Introduction

Linked annotations are a vital element in biomedical data curation. Various tools and options exist to achieve better annotation and curation. The assistive options to help the curators pick proper entities and relations are very important. Many modern annotation tools are based on web technologies [1], and traditionally, there are various server-based solutions to assist in searching, selecting, and autosuggesting interactions in user interfaces. These assistive interactions help the curators and data providers ensure the quality of the annotations and ease the annotation process.

The development of advanced browser engines and browser-oriented lightweight technology stacks have revolutionized the development of web applications, enabling the creation of highly functional interfaces with minimal reliance on server-side processing for some specific use cases. This evolution is particularly relevant in implementing assistive linked annotations for data entry- and edit-interfaces with a notion that an efficient and responsive UI/UX is essential for better annotation support for the curators and data providers.

Since the introduction of ES6, the ECMAScript/JavaScript ecosystem has become increasingly powerful and efficient. This has also prompted the creation of modern type-safe languages like TypeScript and the development of various web frameworks. These advancements and widespread adoption have paved the way for modern JavaScript runtimes such as Node.js, Deno, Bun, LRT, and WinterJS. Platforms like Cloudflare Workers and Deno Deploy have enabled the efficient execution of serverless functions, making JavaScript and WebAssembly-based solutions more appealing for a wide range of applications.

From a developer’s perspective, these improvements have enhanced developer experience (DX) and facilitated the creation and deployment of applications and API services using lightweight technology stacks, including JavaScript and WebAssembly. The growing popularity of edge computing and serverless functions has led to continual performance and cost-efficiency improvements. Consequently, building more powerful and cost-effective biomedical annotation and visualization tools and services is now possible [2]. As full-stack solutions, these lightweight technology stacks offer high portability across web interfaces, servers, serverless functions, edge computing platforms, and even desktop and mobile applications.

This report documents the process and outcomes of a hackathon project designed to test and explore these concepts by creating a proof-of-concept implementation.

### Objective and rationale

The primary objective of this project is to explore the feasibility of using lightweight technology stacks oriented toward web browsers and browser engines, such as JavaScript and WebAssembly (WASM) implementations, to enhance assistive linked annotations. Specifically, implementing in-browser Named Entity Recognition (NER), serverless embedding and vector search within web interfaces, and efficient serverless full-text search. By leveraging edge functions and modern JavaScript frameworks, this hacking project aimed to provide a proof of concept demonstrating these technologies’ usability and potential benefits.

Lightweight technology stacks present several compelling use cases in the context of assistive linked annotations, particularly within the medical and clinical fields where privacy, security, and real-time performance are critical. One significant application is Medical Record Annotation in Restricted Environments. Due to privacy and security concerns, network access is limited or highly regulated in many medical settings. However, the need to annotate and structure patient information, such as extracting symptoms from clinical records using the Human Phenotype Ontology (HPO), remains essential. Traditional annotation tools that rely on server communication are unsuitable in such environments. Lightweight, client-side technology stacks enable these tools to function entirely on the client side, providing a local-first, privacy-oriented solution that adheres to stringent data protection regulations, ensuring that sensitive information remains secure. In clinical research, researchers often operate in environments without reliable internet access, such as field hospitals or rural clinics. Client-side annotation tools, built with lightweight technology stacks, allow these researchers to continue their work without interruption. Once network access is available, data can be synchronized with central systems, enabling seamless integration into larger research workflows.

Real-time Patient Data Annotation is another critical use case. Healthcare professionals can leverage lightweight annotation tools to tag quickly and structure data during patient consultations. This ensures that medical records are updated accurately and promptly, without the delays caused by network latency, which is particularly important in time-sensitive clinical environments. Additionally, lightweight technology stacks offer significant advantages in scenarios where Privacy-Sensitive Data Annotation is required, such as annotating genetic data or sensitive medical histories. By processing data entirely on the client side, these tools eliminate the need for information to leave the local environment, significantly reducing the risk of data breaches and ensuring compliance with privacy regulations. It improves privacy and security by eliminating the need for server-side data processing and storage.

Beyond these specific use cases, lightweight annotation stacks offer general benefits for enhancing annotation interfaces. They can provide quick suggestions to annotators, speeding up the annotation process and avoiding significant delays associated with server communication. By leveraging client-side processing, we can significantly reduce latency—the time delay between the cause and its observed effect—thereby providing a more responsive and user-friendly interface [3]. By offloading many of these tasks to the client side, these tools achieve faster performance than traditional client-server architectures, dramatically reducing latency and improving the overall user experience. Also, client-side processing enhances cost efficiency by reducing the reliance on server resources, making the system more economical.

### Use cases

A few use cases where lightweight technology stacks can be used to enhance assistive linked annotations are:

- Develop tools that run entirely on the client side, eliminating the need for server-side processing and allowing users to annotate data without a network connection.
- Implement API services that leverage edge computing to process requests closer to the user, reducing latency and improving response times.
- Create responsive and user-friendly UI components for annotation tools, enhancing the user experience.
- Design tools prioritizing privacy and security by avoiding server-side processing and data storage, ensuring that all data remains on the client side.
- Develop tools that provide real-time assistive hints to annotators, using client-side processing to ensure immediate feedback.
- Build portable and efficient tools, capable of running on various run-times and platforms, ensuring broad compatibility and ease of deployment.
- Create standalone desktop and mobile applications using frameworks such as Tauri, Electron, and React Native, offering robust and versatile annotation solutions across different devices.
- Use client-sided vector search to generate retrieval-augmented generation (RAG) based LLM prompts to enhance the interfaces and annotation workflows further [4].

## Methods

A proof-of-concept web application was developed to demonstrate the feasibility and performance of lightweight technology stacks for assistive linked annotations. The demonstration provides the Cell Ontology [5], an ontology from the OBO Foundry that encompasses the domain of biological cell types and primarily focuses on animal cell types, and the Human Phenotype Ontology (HPO), which is widely used in clinical and research settings to represent and categorize human phenotypic abnormalities [6]. The HPO provides a standardized vocabulary for describing phenotypic traits associated with human diseases, facilitating the diagnosis, research, and annotation of genetic and clinical data.

The web application was developed using SvelteKit^1^, a modern JavaScript web framework based on Svelte. The proof of concept (PoC) is composed and themed with Flowbite components for Svelte^2^. WinkJS was utilized to implement the NLP and NER functionalities. WinkJS^3^ is an open-source package suite for natural language processing, statistical analysis, and machine learning in Node.js. This PoC uses the wink-eng-lite-web-model, wink-ner, wink-nlp, and wink-tokenizer packages from the WinkJS project.

To generate the embedding vectors for the entities in the Cell Ontology, the GTE-Small model was used. The GTE-Small model is a lightweight version of the GTE model, a general-purpose text embedding model trained with multi-stage contrastive learning [7]. To implement it in the proof-of-concept web application, Supabase’s fork of the GTE-Small model with ONNX weights was used to be compatible with Transformers.js^4^. The model was loaded and executed in the browser using Transformers.js. Transformers.js^5^ is designed to be functionally equivalent to Hugging Face’s Transformers Python library, enabling the execution of pre-trained models through a similar API. By leveraging ONNX Runtime^6^, Transformers.js allows these models to run directly in the browser.

For the full-text search and vector search functionalities, the PoC web application uses the Orama search library^7^. Orama search is a full-text, vector, and hybrid search engine that runs in the browser, on servers, and at edge platforms. Orama search was selected for its support for both full-text and vector search, allowing a single library to demonstrate both search functionalities. The Orama search library is used to index the ontology entities and their embeddings using the GTE-Small model. The search index is served as static files and loaded in the browser on demand.

**Fig. 1:**
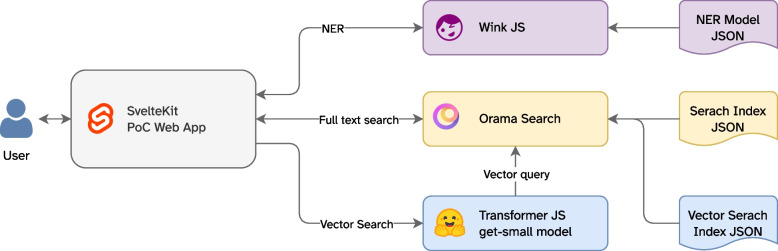
Schematic overview of the proof-of-concept (PoC) web application

A schematic overview of the PoC web application is shown in Fig. 1. The web application consists of three main components: the NER component, the embedding component, and the search component. The NER component is responsible for recognizing named entities in the text. The embedding component generates embedding for the search query using the GTE-Small model. The search component allows the user to search for entities based on their text or embedding vectors.

## Results

A fully functional proof-of-concept (PoC) web application was developed to demonstrate the feasibility of using lightweight technology stacks for assistive linked annotations. To provide concrete evidence that such applications can be deployed as client-side-only applications, the PoC web application was deployed as a static website on GitHub Pages. This deployment is accomplished using GitHub Actions and the SvelteKit adapter for static sites. The web application can be accessed at https://nishad.github.io/lannotate/, and the source code is available at https://github.com/nishad/lannotate.

**Fig. 2:**
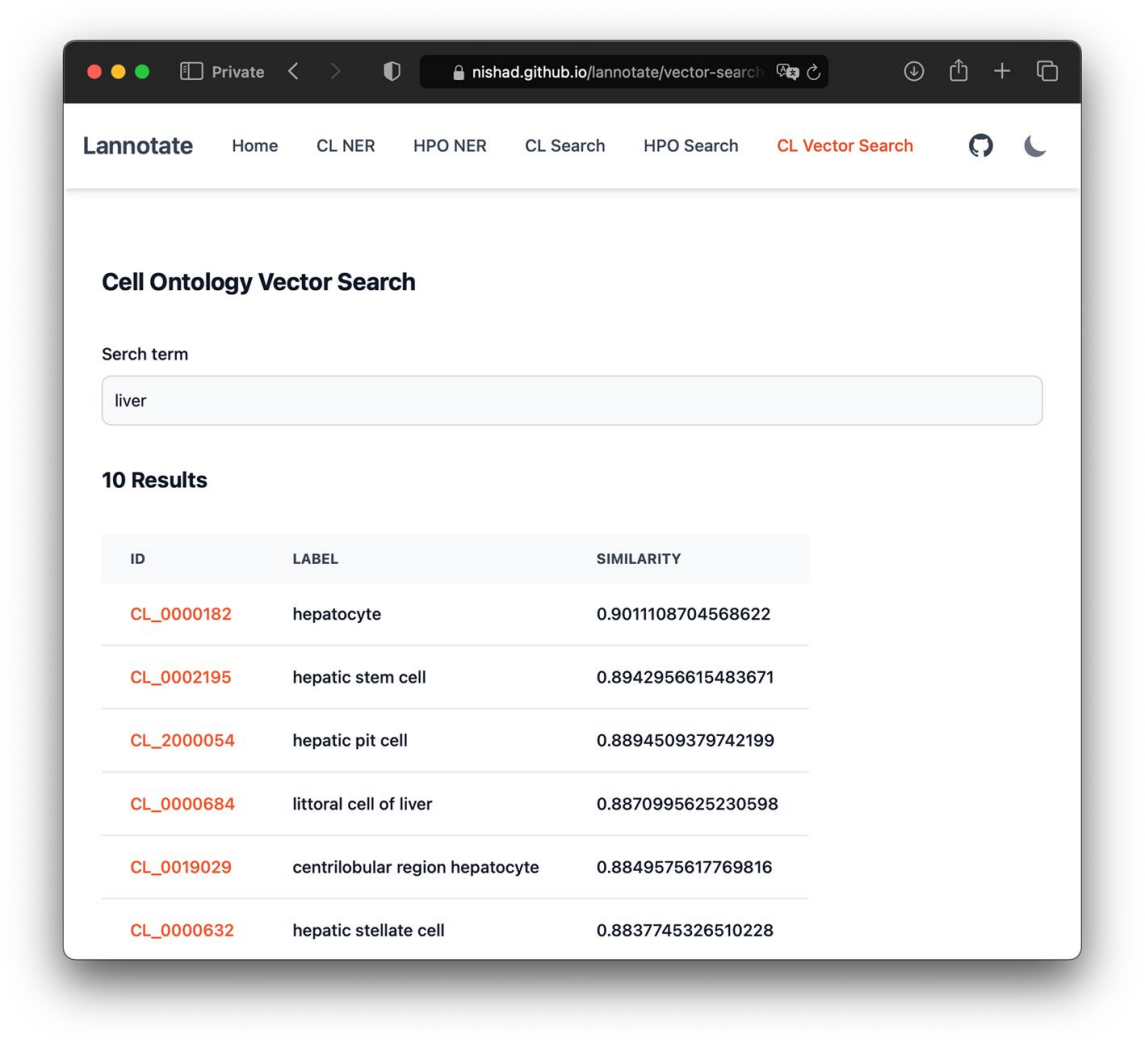
A screenshot of the PoC web application performing a semantic search using the vector search option

The PoC web application was tested across common web browsers and devices to ensure responsiveness and efficiency. The application consistently performed well in all tested environments, demonstrating robustness and low latency response. The resulting PoC successfully showcases the feasibility of implementing in-browser Named Entity Recognition (NER), serverless embedding and vector search, and efficient serverless full-text search. Additionally, the NER component of the PoC web application can generate PubAnnotation [8] TextAE annotation JSON^8^. The Named Entity Recognition (NER) component identifies and highlights key terms like "cell type" or "symptom" within medical records, providing immediate context and support for the annotator. The Full-text Search functionality allows users to retrieve all records containing specific terms. For example, searching for "lymph" might return related concepts like "lymphocyte," "intraepithelial lymphocyte," or "blood lymphocyte." The Vector Search enables semantic querying, which is particularly useful in medical contexts with different terms or synonyms. For example, searching for "liver" might return related concepts like "hepatocyte" or "hepatic cell," even if the exact term is not present in the records, demonstrating the tool’s capability to handle complex, domain-specific queries. Figure 2 shows a screenshot of the PoC web application performing a semantic search using the vector search option.

## Discussions and conclusions

Although the PoC web application demonstrates the feasibility of using lightweight technology stacks for assistive linked annotations, it is not a complete solution for such tools. The PoC serves as an initial step, showcasing the potential of these technologies. However, for each specific use case, there must be dedicated implementation and optimization of lightweight technology stacks to achieve the required performance and efficiency. For large-scale and complex annotation tasks, lightweight technology stacks alone may not be sufficient. In these cases, a hybrid solution incorporating both server-side and client-side processing can be utilized to enhance performance and scalability. The embedding and search functionalities may also need more efficient and optimized implementations for larger and multiple ontologies. Proper evaluation and benchmarking of the selected libraries and implementation strategies should be conducted for real-world applications.

The embedding model used in the PoC is a general-purpose text embedding model. For specific domains and ontologies, such as those in bioinformatics, domain-specific embedding models may be necessary to achieve better search results. Utilizing models tailored to the specific vocabulary and context of the domain can significantly improve the accuracy and relevance of the semantic search results. The PoC selectively utilized specific libraries and tools for demonstration purposes. However, many other libraries and tools can achieve similar functionalities. For example, NLP.js^9^ is another library that can be used for NLP and NER functionalities in the browser. The choice of libraries and tools should be based on the application’s specific requirements and use cases.

One significant challenge with client-side annotation assistive technologies is ensuring that ontologies and controlled vocabularies used for annotation remain up-to-date. In a client-server model, updates to ontologies and terms can be centrally managed on the server, simplifying the process. However, in a client-side setup, each instance of the software must ensure its locally available ontologies are current, which can be more complex to manage across multiple devices. Additionally, after annotations are made, securely synchronizing this data with centralized databases or other systems introduces further challenges. Robust data synchronization and conflict resolution mechanisms are essential, particularly in environments where patient safety and data integrity are critical. While client-side processing offers advantages such as privacy and local data control, it can also lead to performance issues, especially when large ontologies or datasets must be loaded into the browser. For instance, the initial loading of extensive search indexes required for full-text or vector search could cause significant bandwidth consumption and slow performance in web applications. These issues highlight the need for further optimization and hybrid approaches that combine local processing with selective server-side support. Such approaches mitigate the trade-offs and ensure that offline and online scenarios are effectively supported.

While the advantages of utilizing lightweight technology stacks for client-side processing are evident, there are inherent challenges associated with offloading a significant portion of resources to clients. One of the primary concerns is the potential for excessive memory and resource usage on client devices, which can lead to performance degradation or even system instability, particularly in resource-constrained environments.

During the testing of the proof-of-concept (PoC) implementation, it was observed that the heap memory usage remained relatively low, generally below 50 MB. For smaller ontologies, such as Cell Ontology, the memory footprint was even smaller, typically under 25 MB. This demonstrates the efficiency of the PoC under typical conditions, especially when handling lightweight or moderately sized ontologies. However, there was a noticeable increase in memory usage during interactions with the interface, mainly when performing full-text and vector searches. For instance, while executing vector searches, there was a slight uptick in memory and CPU usage. Although this increase was minimal and did not negatively impact performance in the tested scenarios, it raises concerns about scalability. The resource demands could escalate substantially if the system were required to load and process significantly larger ontologies or multiple ontologies simultaneously. In such scenarios, each loaded index could contribute to considerable memory usage, potentially overwhelming the client system. This issue becomes especially critical when considering deploying this technology in environments where high performance and reliability are non-negotiable, such as medical settings. The risk of client-side systems hanging or crashing due to insufficient memory or excessive CPU load must be mitigated through careful engineering and optimization strategies.

Further enhancements are required to address these potential issues. Optimizations could include more efficient memory management, lazy loading of ontologies, and the use of hybrid approaches that offload some of the more intensive processing tasks back to the server. Such measures would ensure that the system remains responsive and reliable, even as the complexity and scale of the ontologies increase.

One feasible solution is to create ontology subsets based on specific profiles to address the challenges associated with loading and processing large ontologies on the client side. These subsets can be tailored for particular annotation purposes, such as in clinical settings where only a relevant portion of a large ontology, like the Human Phenotype Ontology (HPO), is required. By focusing on a specific use case or clinical scenario, the ontology can be subsetted to include only the most relevant terms, significantly reducing the memory footprint and processing requirements on the client. These subsets can be loaded to the client on demand, automatically, or through manual interaction, where the annotator selects the appropriate profile to load. This approach minimizes the bandwidth required for initially loading the indexes and mitigates the performance impact associated with processing extremely large ontologies. It allows the system to remain responsive and efficient, even when dealing with complex and data-intensive tasks.

Another futuristic approach for managing large ontologies on the client side is using Small Language Models (SLM) or Tiny Large Language Models (LLM). These models can potentially automate the loading of additional ontology subsets or related terms as needed, providing a more dynamic and adaptive annotation environment. However, this approach requires further exploration, particularly as APIs for interacting with embedded LLM in modern browsers still need to be finalized^10^. Although these technologies are advancing rapidly, practical implementation and testing are not yet fully accessible, which presents a challenge for immediate deployment.

Upcoming specifications and standards like WebGPU and WebNN promise to provide more efficient and optimized solutions for client-side processing of NLP and NER functionalities. These advancements could further enhance the performance of lightweight technology stacks in handling complex tasks directly in the browser. In the future, more sophisticated and optimized lightweight Large Language Models (LLMs) and Small Language Models will likely become available for client-side execution. These models could significantly improve assistive annotations by offering advanced NLP capabilities without server-side processing.

The PoC web application developed in this study illustrates the potential of lightweight technology stacks for enhancing assistive linked annotations. While the PoC successfully demonstrates the feasibility of implementing in-browser NER, serverless embedding, vector search, and efficient serverless full-text search, it also highlights the need for further development and optimization to create comprehensive solutions for real-world applications. As technology evolves, the prospects for developing robust, responsive, and user-friendly annotation interfaces using lightweight stacks are promising, paving the way for significant advancements in biomedical informatics and beyond.

## Data Availability

The data described in this report can be freely and openly accessed at Zenodo: https://doi.org/10.5281/zenodo.11467006 [9].

## Footnotes

1 https://kit.svelte.dev

2 https://flowbite-svelte.com

3 https://winkjs.org

4 https://huggingface.co/Supabase/gte-small

5 https://huggingface.co/docs/transformers.js/en/index

6 https://onnxruntime.ai/

7 https://askorama.ai

8 https://textae.pubannotation.org

9 https://github.com/axa-group/nlp.js

10 https://developer.chrome.com/docs/ai
